# IrO_2_ Oxygen Evolution Catalysts Prepared by an Optimized Photodeposition Process on TiO_2_ Substrates

**DOI:** 10.3390/molecules29102392

**Published:** 2024-05-19

**Authors:** Angeliki Banti, Christina Zafeiridou, Michail Charalampakis, Olga-Niki Spyridou, Jenia Georgieva, Vasileios Binas, Efrosyni Mitrousi, Sotiris Sotiropoulos

**Affiliations:** 1 Physical Chemistry Laboratory, Department of Chemistry, Aristotle University of Thessaloniki, 54124 Thessaloniki, Greece; zafeirid@ualberta.ca (C.Z.); nikiolga9@gmail.com (O.-N.S.); vbinas@chem.auth.gr (V.B.); mitrouse@chem.auth.gr (E.M.); 2 Institute of Electronic Structure and Laser, Foundation for Research and Technology-Hellas (FORTH-IESL), 70013 Herakleion, Greece; michalisxaral@gmail.com; 3 Rostislaw Kaischew Institute of Physical Chemistry, Bulgarian Academy of Sciences, 1113 Sofia, Bulgaria; jenia@ipc.bas.bg

**Keywords:** iridium nanoparticles, titanium oxide, photodeposition, oxygen evolution reaction

## Abstract

Preparing high-performance oxygen evolution reaction (OER) catalysts with low precious metal loadings for water electrolysis applications (e.g., for green hydrogen production) is challenging and requires electrically conductive, high-surface-area, and stable support materials. Combining the properties of stable TiO_2_ with those of active iridium oxide, we synthesized highly active electrodes for OER in acidic media. TiO_2_ powders (both commercially available Degussa P-25^®^ and hydrothermally prepared in the laboratory from TiOSO_4_, either as received/prepared or following ammonolysis to be converted to titania black), were decorated with IrO_2_ by UV photodeposition from Ir(III) aqueous solutions of varied methanol scavenger concentrations. TEM, EDS, FESEM, XPS, and XRD measurements demonstrate that the optimized version of the photodeposition preparation method (i.e., with no added methanol) leads to direct deposition of well-dispersed IrO_2_ nanoparticles. The electroactive surface area and electrocatalytic performance towards OER of these catalysts have been evaluated by cyclic voltammetry (CV), Linear Sweep Voltammetry (LSV), and Electrochemical Impedance Spectroscopy (EIS) in 0.1 M HClO_4_ solutions. All TiO_2_-based catalysts exhibited better mass-specific (as well as intrinsic) OER activity than commercial unsupported IrO_2_, with the best of them (IrO_2_ on Degussa P-25^®^ ΤiO_2_ and laboratory-made TiO_2_ black) showing 100 mAmg_Ir_^−1^ at an overpotential of η = 243 mV. Chronoamperometry (CA) experiments also proved good medium-term stability of the optimum IrO_2_/TiO_2_ electrodes during OER.

## 1. Introduction

Among water splitting technologies (towards a hydrogen economy [1]), proton exchange membrane water electrolyzers (PEMWEs), especially when coupled with renewable energy sources, have been considered as the most promising and appealing systems for high-purity hydrogen production due to their fast load change, high current density, and energy efficiency [2]. However, the highly corrosive operating potential (E > 1.5 V vs. RHE), the sluggish kinetics of the OER, and the acidic environment (pH < 1) at the anode of a PEMWE cell are the main hindrances for the water electrolysis reaction, and studies have focused on investigating suitable stable and low-overpotential OER catalysts [3]. Among the electrode materials quoted in the literature, and in line with the relevant catalytic activity volcano plots as well as stability issues, only precious metal oxides, especially IrO_2_ and iridium-based oxides, are suitable anode catalysts, as these combine high catalytic activity and long-term stability in the acidic conditions of PEMWEs [4]. Considering the high cost and limited availability of iridium, many efforts have been devoted to preparing a cost-effective OER electrocatalyst with the minimum amount of the noble and scarce metal. This can be achieved by increasing the intrinsic catalytic activity of IrO_2_ as well as its surface area; both challenges can be addressed by the choice of an appropriate catalyst support material [5].

Among various support candidates, TiO_2_ is a promising material both at a pilot scale and for large scale applications, given its low cost, abundance, high stability, corrosion resistance, and mature industry production [6,7]. Despite its poor electrical conductivity and catalytic activity, the decoration of the TiO_2_ oxide support with conducting IrO_2_ nanoparticles as well as the electronic interaction between them form an electrically percolating structure (with a specific conductivity of >0.1 S cm^−1^) with improved overall activity and stability [8,9,10,11,12], in line with theoretical predictions [13].

Preparation methods for IrO_x_/TiO_2_ catalysts include the Adams fusion method [10,12,14,15], thermal treatment in air or H_2_ [11,16,17,18], hydrothermal reactions [19,20], and electrodeposition [21,22]. Photocatalytic deposition of IrO_x_ on semiconductors is neither a common practice nor a straightforward process from a mechanistic point of view (it usually involves photolytic hydrolysis of Ir salts), among the number of methods reported for IrO_x_ preparation [23]. There have been very few publications on Ir or IrO_x_ photodeposition on semiconductor substrates from Ir(III) or Ir(IV) solutions [24,25,26,27] and a single one from Ir(III) complexes onto TiO_2_ photoanodes [27]. We have recently photodeposited Ir/IrO_x_ from Ir(III) solutions onto TiO_2_ using methanol as a hole scavenger and testing the resulting materials as a catalyst for OER in the dark [28]. However, the role of methanol and the mechanism of photodeposition remained unclear; commercial Degussa P25^®^ was the only substrate used and the resulting catalyst did not outperform the commercial IrO_2_ catalyst for OER.

The aim of the work presented here has been to decipher the mechanism of IrO_x_ photodeposition onto TiO_2_, explore the use of various TiO_2_ substrates, and optimize the OER performance of the thus prepared IrO_x_/TiO_2_ material. To that direction, (a) we have studied the effect of methanol on Ir deposition; (b) we have used four different TiO_2_ supports: Degussa P25^®^ TiO_2_, laboratory-made TiO_2_ from titanium oxysulphate, and their titania black analogs prepared by ammonolysis (seeking alternative, more conducting substrates); and (c) we have tested the materials’ catalytic performance towards OER by CV, LSV, EIS, and CA electrochemical techniques. Hence, the main novelty of this work is unraveling the mechanism of Ir/IrO_x_ photodeposition on TiO_2_ and preparing catalysts that can outperform the state-of-the-art Ir-based OER catalysts, in terms of Ir mass-specific activity.

## 2. Results

### 2.1. Structural, Chemical, and Morphological Characterization of TiO_2_ Materials

**Figure 1:**
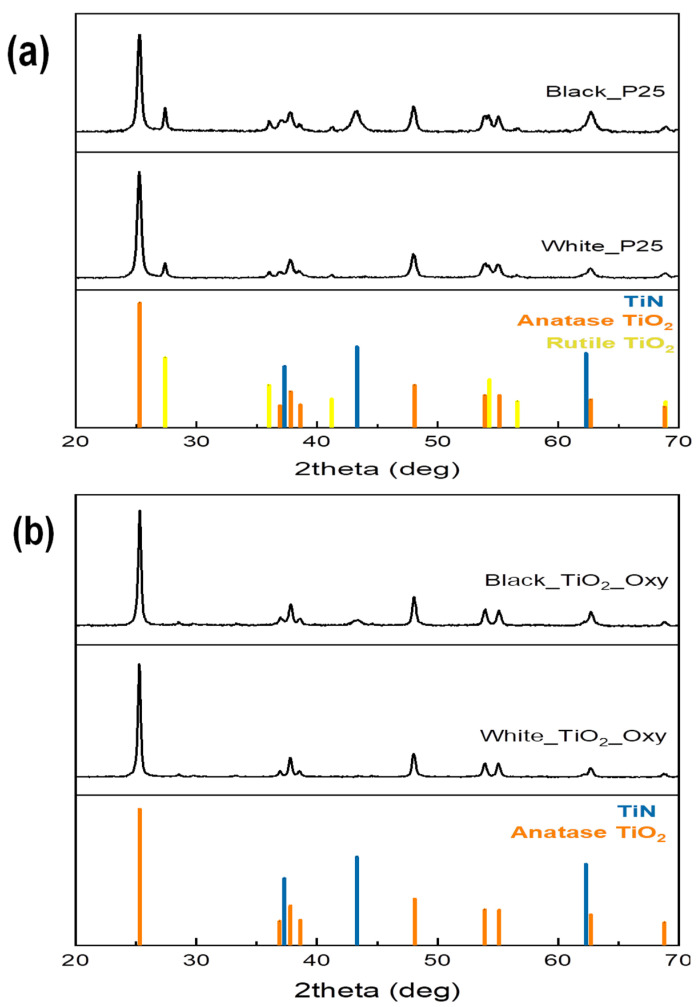
XRD patterns of (**a**) TiO_2_ P25^®^ and (**b**) TiO_2_-oxy, before (“white”) and after ammonolysis (“black”), respectively.

XRD patterns were recorded for commercial Degussa P25^®^ TiO_2_ (“white-P25” hereafter), black TiO_2_ P25^®^ produced by ammonolysis (see Section 4 below) (“black-P25” hereafter), laboratory-made TiO_2_ from titanium oxysulfate (“white-TiO_2_-oxy” hereafter), and black TiO_2_-oxysulfate nanoparticles also produced by ammonolysis (see Experimental below) (“black-TiO_2_-oxy” hereafter). These are illustrated in Figure 1a,b. Diffraction peaks at 2θ values of 25.3°, 36.9°, 48.1°, 53.9°, 55.1°, and 62.7° can be assigned to (101), (004), (200), (105), (211), and (116) planes of the anatase phase. The additional peaks at 27.4°, 36°, 41.2°, 54.3°, 56.6°, and 68.9° can be ascribed to (110), (101), (111), (211), (220), and (301) planes of the rutile phase. TiO_2_ P25^®^ nanoparticles consist of a mixture of anatase and rutile phases as expected for the much-studied commercial material. In contrast, for the laboratory-prepared TiO_2_-oxy catalysts, the ΤiO_2_ rutile phase is not present. The intensity of all these peaks before and after ammonolysis is stable, which means that the structure of TiO_2_ remains the same in both their “white” and “black” forms. Using the Scherrer equation, the average TiO_2_ crystallite size was estimated to be 21.2 and 23.1 nm for white-P25 and black-P25, respectively, and 29.6 nm and 28.5 nm for white-TiO_2_-oxy and black-TiO_2_-oxy, respectively (i.e., the laboratory-made materials have larger crystallites). 

The only significant change in the XRD diffractograms after the ammonolysis process is that both materials show new rutile peaks which correspond to the formation of titanium nitride (TiN); these peaks appear at 2θ values of 37.3°, 43.3°, and 62.3° and they can be indexed to (111), (200) and (220), planes.

**Figure 2:**
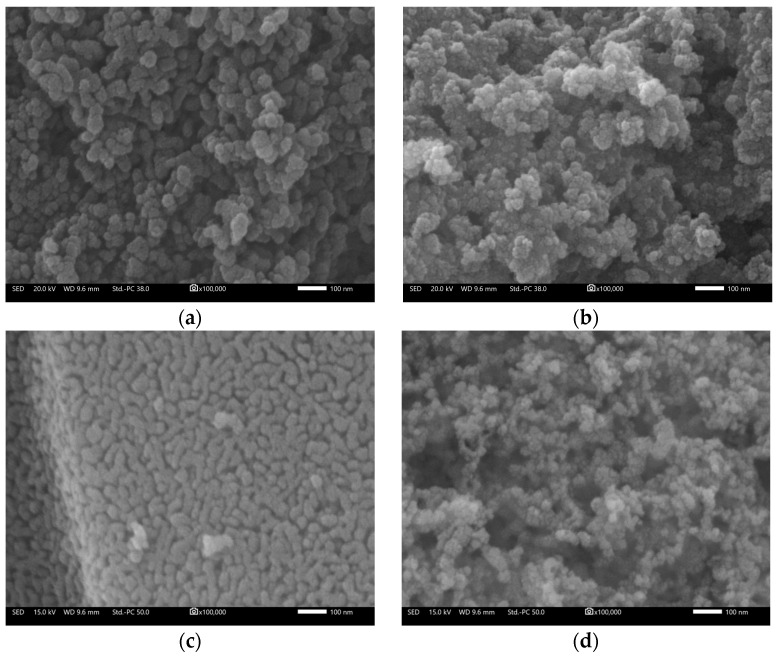
FESEM images of (**a**,**b**) white-P25 and black-P25 and (**c**,**d**) white-TiO_2_-oxy and black-TiO_2_-oxy (scale bar corresponds to a length of 100 nm).

The morphology of the TiO_2_ powders can be seen in the FESEM images of Figure 2 below. White-P25 (Figure 2a) and black-P25 (Figure 2b) consist of nanoparticles with diameters around ~20 nm and have similar morphology (in black-P25, nanoparticles are slightly more agglomerated). White-TiO_2_-oxy (Figure 2c) consists of nanoparticles (~30 nm) that aggregate into rod-like structures which change into smaller nodular particles and rougher aggregates upon their transformation to black-TiO_2_-oxy (Figure 2d). The nanoparticle size agrees with calculations from the XRD data, using the Scherrer equation. 

In addition, elemental analysis (Table 1) and EDS mapping confirms the presence of titanium and oxygen in the appropriate ratios for these oxides as well as the presence of nitrogen in the ammonia-treated samples, due to TiN formation.

**Figure 3:**
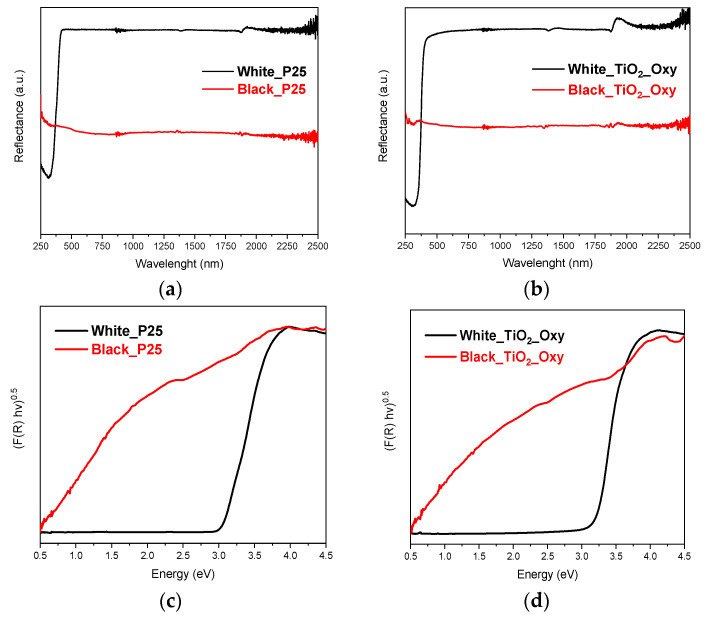
UV-vis diffuse reflectance spectra of TiO_2_ nanoparticles ((**a**): P25; (**b**): TiO_2_-oxy) and corresponding Tauc plots ((**c**): P25; (**d**): TiO_2_-oxy).

The diffuse reflectance spectra (DRS) of TiO_2_ nanoparticles before and after ammonolysis are shown in Figure 3a,b (for P25 and TiO_2_-oxy samples, respectively), with the corresponding Tauc plots in Figure 3c,d. The former plots confirm that the “black” catalyst variants absorb light in the visible range too. The optical band gaps of the commercial (P25) and synthesized (TiO_2_-oxy) photocatalysts were estimated using the Tauc method, as described in Section 4 below. For white-P25, this can be estimated at 3.06 eV and can be attributed to the mixture of anatase (3.2 eV) and rutile (3.0 eV) phases of TiO_2_ (Figure 3c); for white-TiO_2_-oxy, it is estimated at 3.21 eV, which agrees with the anatase phase of TiO_2_. Although for the “black” variants the Tauc plots cannot lead to accurate band gap values, these can be approximated to ≤1 eV for black-P25 and ca 1.5 eV for black-TiO_2_-oxy. The significant decrease in the band gap of ammonia-treated samples should also lead to a substantial increase in their electronic conductivity. The maximum absorbance of white-TiO_2_ and white-TiO_2_-oxy at 365 and 300 nm, respectively, means that UV illumination is mandatory for Ir/IrO_x_ photodeposition. On the contrary, the absorbance of ammonia-treated (“black”) samples means that photodeposition could, in principle, take place under visible light illumination too (however, this has not been possible).

### 2.2. Structural, Chemical, and Morphological Characterization of IrO_x_/TiO_2_ Materials

The XRD patterns of IrO_x_/TiO_2_ modified samples did not show well-defined peaks of crystalline IrO_2_ and were practically identical to those of Figure 1, pointing to the catalyst being deposited in its amorphous form. (It should be noted that literature data [29,30] indicate that IrO_x_ catalysts are more active for OER if the material crystallinity is low or intermediate.).

The morphology of the electroactive IrO_x_ particles is expected to have an important influence on their surface area and on their intrinsic catalytic activity (via their interaction with the substrate) as well as, in this case, on electronic conductivity (since the latter is expected to depend on interparticle contacts). TEM analysis was performed to characterize the morphology of the IrO_2_/TiO_2_ electrocatalysts. 

**Figure 4:**
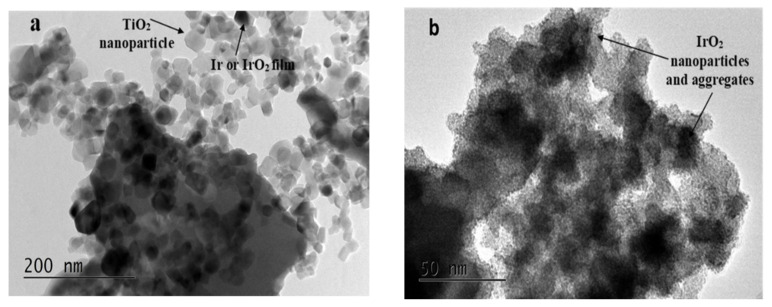
TEM images of (**a**) the photocatalytically prepared powder IrO_2_/TiO_2_ (white-P25^®^) and of (**b**) commercial IrO_2_ (scale bars correspond to a length of 200 nm (**a**) and 50 nm (**b**)).

Figure 4a shows the morphology of the photocatalytically prepared powder of IrO_2_/TiO_2_ (white-P25^®^), while Figure 4b depicts that of the commercial IrO_2_ catalyst (Alfa Aesar, Karlsruhe, Germany). TEM micrographs provide a first indication of the successful photodeposition of Ir or IrO_2_ particles/films (26% *w*/*w* Ir, as further confirmed by EDS) onto the TiO_x_ particles (the latter having a ca 30 nm nominal size in the case of white-P25^®^). Ir or IrO_x_ can be seen as black areas, partly covering the TiO_2_ particles, in a non-uniform manner. On the other hand, unsupported commercial IrO_2_ nanoparticles (of 2–3 nm nominal size) tend to form aggregates of different sizes.

**Figure 5:**
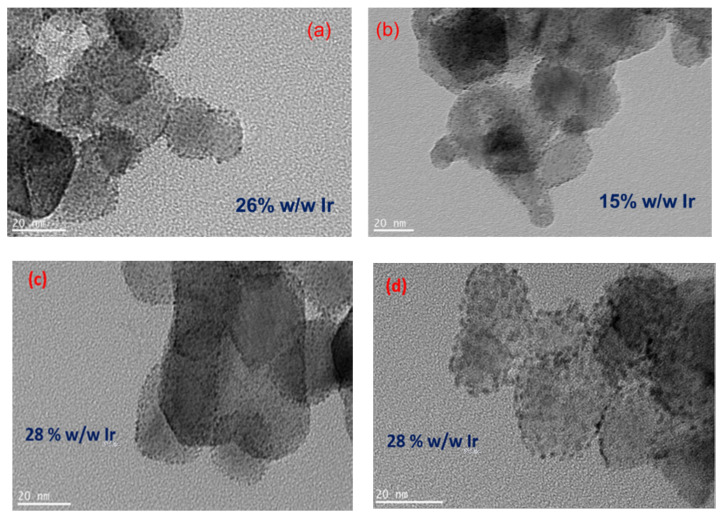
TEM micrographs of (**a**) IrO_x_/TiO_2_ (white-P25), (**b**) IrO_x_/TiO_2_ (black-P25), (**c**) IrO_x_/TiO_2_ (white-TiO_2_-oxy) and (**d**) IrO_x_/TiO_2_ (black-TiO_2_-oxy) (scale bar corresponds to a length of 20 nm).

Higher-resolution TEM images of all the obtained catalysts are shown in Figure 5a–d and a uniform dispersion of Ir/IrO_x_ nanoparticles on the larger TiO_2_ support particles can be seen. Such a good conducting Ir/IrO_x_ particle dispersion is crucial for providing good interparticle contact and electronic conductivity. The energy-dispersive spectroscopy (EDS) of the samples shows the existence of Ir—more specifically, for all catalysts, a ca 26 *w*/*w* % Ir content, except for black-P25 with a 15 *w*/*w* % Ir content.

**Figure 6:**
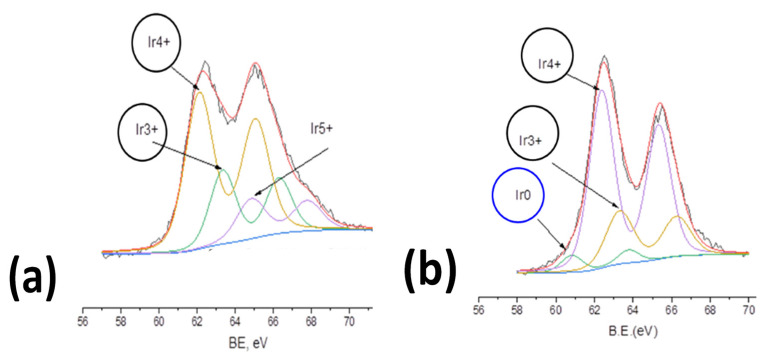
X-ray photoelectron spectra in the Ir4f binding energy range of IrO_x_/TiO_2_ (white-P25) powder nanocatalyst prepared by UV photodeposition in the absence (**a**) and presence (**b**) of 0.15% *v*/*v* MeOH.

The chemical state of Ir in the TiO_2_-supported catalyst was examined by XPS analysis for catalysts prepared in the presence and absence of methanol (Figure 6). The Ir4f peak was used as a first approach for chemical state identification. Measurements at the 4f band of the sample resulted in peak positions of 65.4 eV and 62.4 eV for the Ir 4f5/2 and Ir 4f7/2 peaks, respectively. These values are close to those reported for hydrated amorphous iridium dioxide, which also correlates with the XRD results. 

Fitting the data to a curve resulting from the convolution of peaks corresponding to the various oxidation states of Ir proves that the catalyst prepared in pure water (Figure 6a) consists almost exclusively of iridium oxides (mainly iridium dioxide-Ir(IV)). However, there is some metallic Ir (Ir(0)) when MeOH is added to the preparation solution (Figure 6b) since the anodic deposition route (Ir(III) oxidized by photogenerated holes) may be partially suppressed (as MeOH competes for holes) and the cathodic deposition route (Ir(III) reduced to Ir by photogenerated electrons) may start to operate too (see also Section 3 below).

### 2.3. Electrochemical Performance

**Figure 7:**
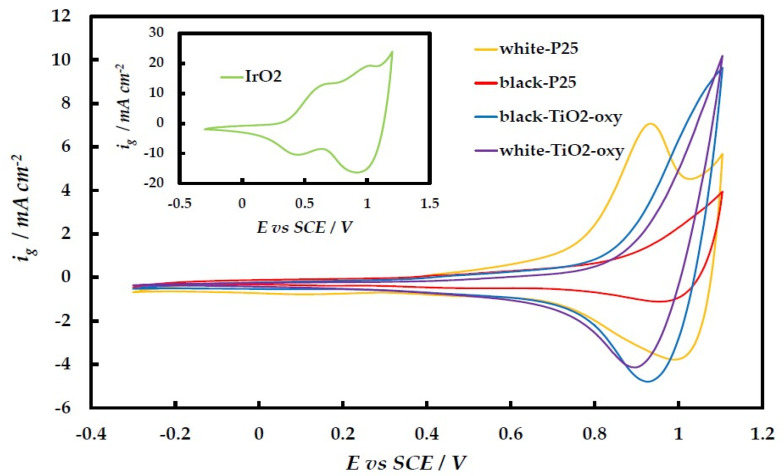
Cyclic voltammetry curves of IrO_x_/TiO_2_ electrodes as well as of commercial IrO_2_ electrode (inset), at 25 mV s^−1^, in a deaerated 0.1 M HClO_4_ solution. Current density, i_g_, is reported per geometric glassy carbon electrode current collector area.

IrO_x_ surface electrochemistry was studied by cyclic voltammetry (CV) experiments in the potential range between hydrogen and oxygen evolution (−0.30 V to +1.10 V vs. SCE) at a 25 mV s^−1^ potential sweep rate. Figure 7 shows the stabilized CV curve for each catalyst (prepared in the absence of methanol). On the anodic part of the CV curve of the commercial, unsupported IrO_x_ catalyst (Inset to Figure 7), one can clearly observe two peaks corresponding to the transformation of Ir(III) to Ir(IV) and Ir(IV) to Ir(V), typical of IrO_x_ surface electrochemistry of thermally or electrochemically (by anodization) prepared IrO_x_ catalysts [29,30]. On the other hand, the photochemically prepared catalysts (main Figure 7) show only one oxidation peak (at ca 0.9 V, corresponding to the Ir(IV) to Ir(V) transformation), pointing to the initial photodeposition mainly of Ir(IV) as IrO_2_ on the TiO_2_ support, which can only be further oxidized to Ir(V) (see also Discussion below). Also, the electrochemically active surface area of the prepared catalysts is lower than that of the commercial one (for similar Ir loadings in the 0.5–1 mg cm^−2^ range). Given the similar particle morphology of the commercial and prepared IrO_x_ particles (see Figure 5), this difference may be attributed to the TiO_2_ substrate, which cannot provide sufficient electronic conductivity between all IrO_x_ nanoparticles. Only those IrO_x_ nanoparticles that are in contact with each other and eventually with the glassy carbon (GC) electrode current collector can act as electroactive material.

**Figure 8:**
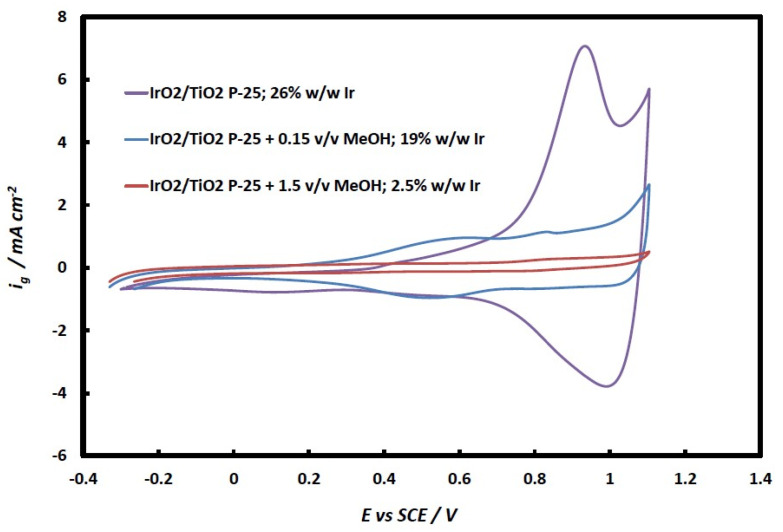
Effect of MeOH content (0, 0.15, 1.5% *v*/*v*) during catalyst preparation by photodeposition onto TiO_2_ from Ir(III) chloro-complex solutions to Ir/IrO_2_ surface electrochemistry.

Figure 8 depicts the effect of methanol used in catalyst preparation based on surface electrochemistry. An absence of methanol (the purple line) leads to higher iridium content (26 *w*/*w* % as confirmed by EDS) and thus a higher electroactive area and dominance of the Ir(IV) to Ir(V) peak. The suppression of iridium deposition when methanol is added can be inferred by the suppression of Ir surface electrochemistry (and is confirmed by EDS results that give 19% and 2.5% *w*/*w* Ir in the presence of 0.15% and 1.5% *v*/*v* MeOH). This is additional proof that IrO_x_ is formed by Ir(III) oxidation by photogenerated holes at the TiO_2_ valence band where MeOH also competes for these photogenerated holes. (Note also that, in the case of 0.15% *v*/*v* MeOH (green line curve), there appears an oxidation wave at ca. +0.5 V corresponding to the Ir(III) to Ir(IV) transformation, indicative of the presence of some metallic Ir too, which is initially converted to Ir(III).).

**Figure 9:**
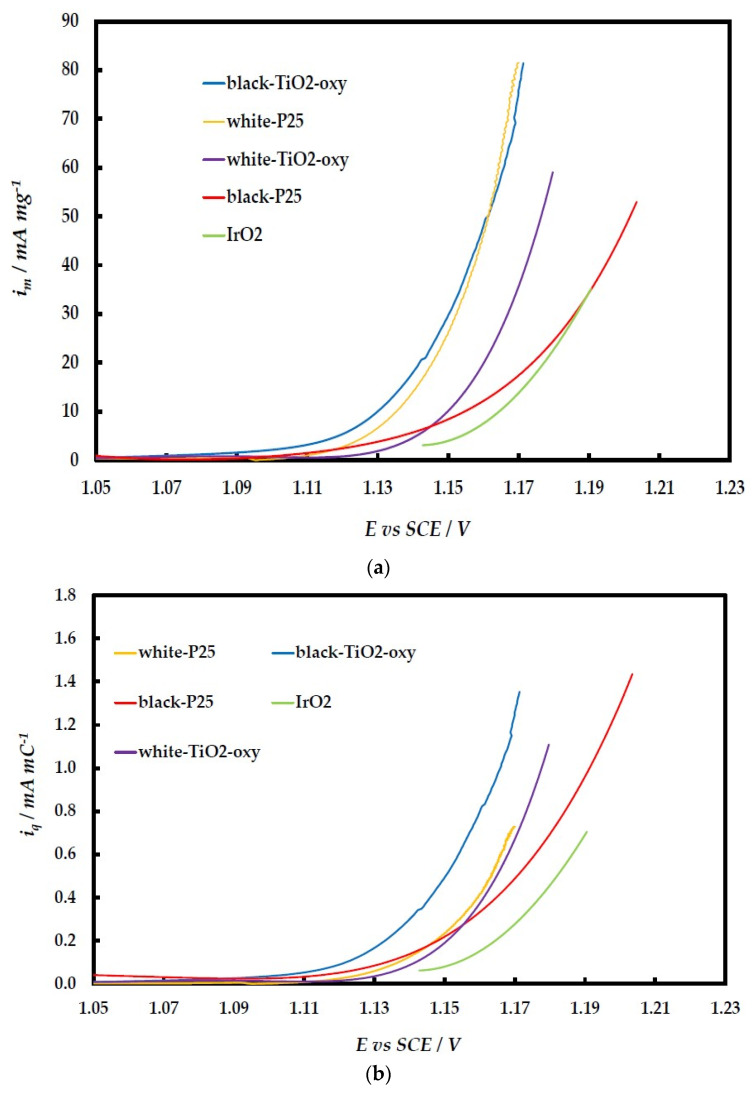
LSVs at 1600 rpm and 5 mVs^−1^ (**a**) per mg of Ir and (**b**) per mC of Ir(IV) to Ir(V) transformation. The potential has been corrected for IR-drop.

Near-steady-state Linear Sweep Voltammetry (LSV) at a slow potential sweep rate (5 mV s^−1^) was employed to investigate the OER activity of the Ir-based catalysts. In Figure 9, currents, i_m_, are reported per Ir mass, and all prepared catalysts (except for those supported on black-P25, bearing the lower Ir content and hence lower conductivity) show improved mass activity compared to the commercial IrO_2_ catalyst. This means that a higher intrinsic catalytic activity of the prepared catalysts (because of strong IrO_2_ and TiO_2_–support interactions) offsets their lower electroactive surface area (because of their lower electronic conductivity and catalyst utilization). This is further confirmed by the LSVs of Figure 9, whereby the OER currents are normalized per the charge corresponding to the 1e^−^ transformation of Ir species, i.e., per its electroactive surface area, resulting in a current descriptor, *i_q_*, that should be independent of surface area and catalyst utilization (e.g., by conductivity restrictions). The intrinsic catalytic activities of IrO_2_ onto white-P25, black-P25, and black-TiO_2_-oxy are almost identical and higher than that of IrO_2_ on white-TiO_2_-oxy and commercial unsupported IrO_2_. A mismatch between Ir and Ti size within their oxide network may destabilize Ir-OH bonds (a reactive intermediate of OER) and increase OER activity.

**Figure 10:**
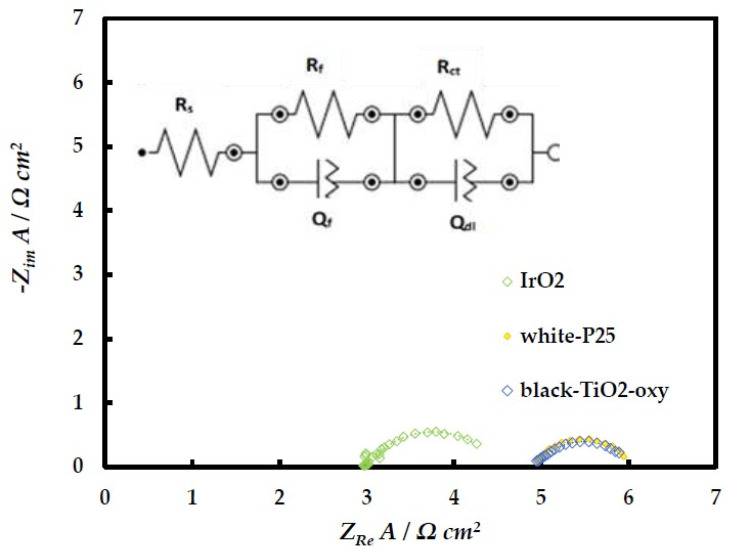
Nyquist plots of IrO_2_-based catalysts at 1.2 V.

Electrochemical Impedance Spectroscopy measurements were performed at 1.2 V in the frequency range of 100 kHz to 100 mHz for the best catalysts according to LSV screening (of Figure 9 above). The experimental data are presented as Nyquist plots in Figure 10 and were fitted to the equivalent electrical circuit R_s_[R_f_Q_f_][R_ct_Q_dl_] (inset of Figure 10). R_s_ corresponds to the ohmic losses (due to the ionic resistance of the solution between the reference and working electrodes, as well as the electronic resistance of the dispersed IrO_2_ electrode); R_f_Q_f_ accounts for the characteristics of ionic transport through the porous electrode and R_ct_Q_dl_ to the OER charge transfer process and the constant-phase element of the IrO_2_/electrolyte interface. (Q_dl_ is transformed to double-layer capacitance C_dl_ using the Mansfield or Brug equations [31,32].) It has recently been argued that low R_ct_C_dl_ values mean high intrinsic electrocatalytic activity [33]. 

Table 2 below shows the results of equivalent circuit analysis, from which it follows that the intrinsic catalytic activity of the IrO_2_/TiO_2_ catalysts towards OER is higher than that of commercial IrO_2_, as the former are characterized by lower R_ct_C_dl_ values. 

**Figure 11:**
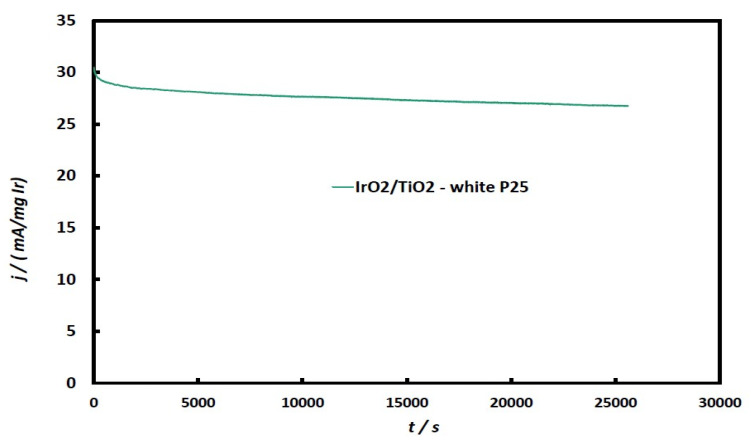
Chronoamperometric curves of the IrO_2_/TiO_2_ (white-P25) electrode, in a deaerated 0.1 M HClO_4_ solution, at a constant potential of +1.3 V vs. SCE, for 7 h (not corrected for IR-drop).

Finally, to confirm the medium-term stability of this type of nano-composite catalyst, after their preliminary electrochemical testing, they were systematically investigated through chronoamperometry experiments at constant potential, and indicative results are shown in Figure 11. It can be seen that catalyst performance retains ca 90% of its initial OER activity during a 7 h experiment.

## 3. Discussion

### 3.1. Analysis of Possible Photodeposition Routes and Mechanisms for Ir Deposition onto TiO_2_ from Ir(III) Solutions

**Figure 12:**
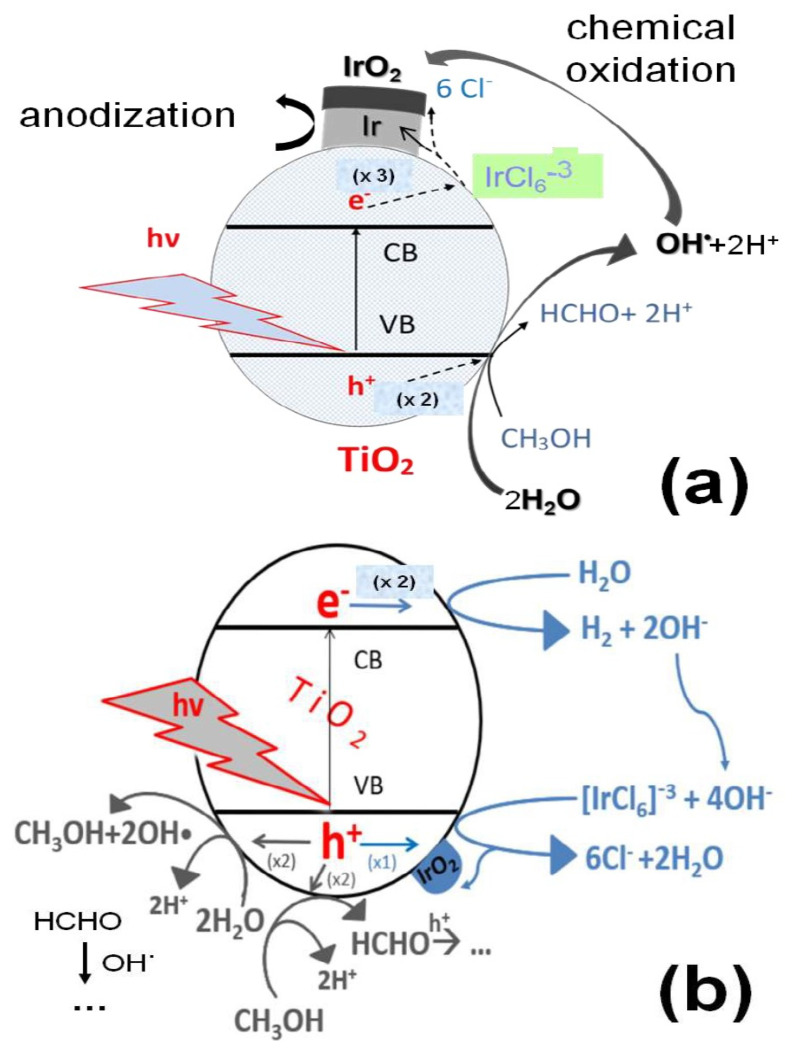
Photodeposition routes of Ir(III) on TiO_2_; (**a**) cathodic deposition of Ir at CB and (**b**) anodic deposition of IrO_2_ at VB. Methanol reactions at VB are also considered.

Being in an intermediate valence state between (0) and (IV), Ir(III) can either be photodeposited as metallic Ir or IrO_2_. The first route involves the cathodic deposition of Ir at the Conduction Band (CB) of the illuminated TiO_2_ semiconductor particles, whereby Ir(III) is photo-reduced to metallic Ir(0) by the photogenerated electrons at the Conduction Band (CB) (Figure 12a); this may be oxidized to IrO_2_ during subsequent electrochemical potential cycling to sufficiently positive potential values (anodization) [28] or chemically oxidized by the OH^·^ radicals resulting from water photo-oxidation. In this case, the presence of methanol (in addition to water as a hole scavenger) should help the Ir(0) deposition process, minimizing electron–hole recombination. The second route (Figure 12b) involves the anodic deposition of IrO_2_ at the valence band (VB), where Ir(III) is photo-oxidized to Ir(VI) by photogenerated holes (or OH^·^) and, in the presence of hydroxyl anions (either in the bulk of an alkaline solution or photo-produced at the CB of the illuminated semiconductor by water photo-reduction), can be chemically converted directly to IrO_2_. In this case, methanol may limit IrO_2_ deposition as it competes for the photogenerated holes (or OH^·^). Irrespective of the photodeposition route followed, and in accordance with the literature [23,27], the first step is reported to be the photo-assisted hydrolysis of the Ir(III) chloro-complex (Equations (1) and (2)):

IrCl_6_^−3^ (UV) → Ir^3+^ (aq) + 6 Cl^−^ (1)

e.g., IrCl_6_^−3^ + 6OH^−^ (UV) → Ir(OH)_6_^−3^+ 6 Cl^−^ (2)

**Figure 13:**
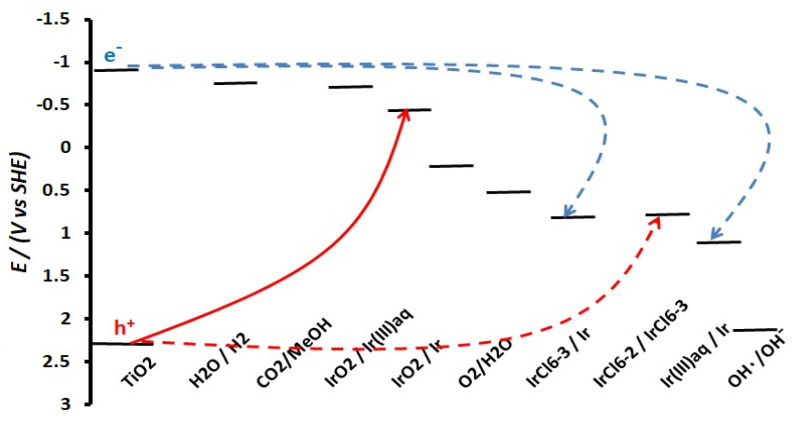
Energy diagram for TiO_2_ (VB and CB) and various ox/red couples.

Predictions for the most favorable processes from a thermodynamic point of view can be made by studying the energy diagram (Figure 13) of TiO_2_ (VB and CB energy levels for the given pH, deduced from data in [34,35]), as well as of the relevant red/ox couples (estimated from data in [36] to account for a pH of 11 and the Ir(III) concentration of 2 × 10^−3^ M). The higher the potential difference, |ΔΕ|, between the energy levels of the TiO_2_ photogenerated holes and electrons and that of a red/ox couple, the more likely the oxidation/reduction reaction, respectively. From the four possible reactions that the red/ox species shown in Figure 13 can undergo (two oxidations (red lines); two reductions (blue lines)), the oxidation of Ir(III)_aq_ to IrO_2_ (|ΔΕ| = 2.77 V) is the most favorable, followed by its reduction to metallic Ir ((|ΔΕ| = 2.01 V):

Ir^3+^ (aq) + h^+^ + 4OH^−^ → IrO_2_ + 2H_2_O (3)

e.g., Ir(OH)_6_^−3^ + h^+^ → IrO_2_ + 2H_2_O + 2OH^−^ (4)

or

Ir^3+^ (aq) + 3e^−^ → Ir (5)

e.g., Ir(OH)_6_^−3^ + 3e^−^ → IrO_2_ + 6OH^−^ (6)

### 3.2. Evidence for the Photodeposition Mechanism and Form of Ir onto TiO_2_ from Ir(III) Solutions

The interpretation of the spectroscopic and electrochemical results presented in the Results section above points to the photodeposition of Ir onto TiO_2_ mainly as IrO_2_, i.e., according to the reaction scheme of Figure 12 and reactions (3) and (4) above.

A first indication that this is the case is given by the effect of methanol addition on Ir content as measured by EDS: this falls from 26% *w*/*w* Ir in the absence of methanol to 19% and 2.5% *w*/*w* Ir upon addition of small quantities of methanol (0.15% and 1.5% *v*/*v* MeOH, respectively). As methanol is expected to compete with Ir(III) for the oxidizing photogenerated species (h^+^ and/or OH^·^), it is expected to adversely affect Ir photodeposition, as has indeed been the case.

Second, the surface electrochemistry of Ir oxides and hydroxides depicted in the CV curves of Figure 7 and Figure 8 shows a single redox peak in the case of catalysts prepared in the absence of methanol, at potentials where Ir(IV) oxide is known to be inter-converted to Ir(V) oxide, without the presence of additional Ir(III)/Ir(IV) redox peaks (as in the case of the commercial catalyst and catalysts prepared in the presence of methanol), whereby Ir(III) would originate from the electrochemical oxidation of metallic Ir to Ir(OH)_3_ at lower applied potentials. 

Finally, XPS confirmed that photodeposited Ir was mainly in its Ir(IV) oxidation state (with its percentage diminishing upon methanol addition).

### 3.3. Electrochemical Performance of the TiO_2_-Supported IrO_2_ towards OER

The surface electrochemistry of Ir oxides/hydroxides of the laboratory-prepared samples (depicted in the CV curves of Figure 7) allows for an estimate of the charge associated with the Ir(IV)/Ir(V) one-electron transformation (by integration of the single anodic peak/wave recorded in the 0.9–1.1 V potential range); this charge, q_IrOx_, is in turn an indicator of the electrocatalyst electroactive surface area (esa). Both the esa and q_IrOx_ of IrO_x_/TiO_2_ catalysts are expected to depend on IrO_x_ quantity, specific surface area dispersion, and conductivity–IrO_x_ inter-connection, while their mass-specific variants (i.e., esa and q_IrOx_ per unit mass of IrO_x_, esa and q_m,IrOx_) should depend only on the latter two parameters. Table 3 below presents the values of mass-specific IrO_x_-charge; in the case of the commercial, unsupported IrO_x_ catalyst (inset of Figure 7), since there are two couples of redox peaks/waves, corresponding to the 2e transformation of Ir(III) to Ir(V) via an In(IV) intermediate, half of the corresponding charge was used for comparison.

It can be seen that the commercial catalyst has the highest mass-specific esa, despite the fact that, according to the TEM results of Figure 4 and Figure 5, its aggregates are larger than the well-dispersed nanoparticles of the laboratory-made catalyst. This means that the decrease in conductivity/interparticle contact of the IrO_x_/TiO_2_ system (due to the semiconductor character of TiO_2_) offsets their higher surface area.

Despite the apparent decrease in their esa, all of the prepared IrO_2_/TiO_2_ catalysts show better mass-specific electrocatalytic activity towards the OER than the commercial unsupported IrO_2_ catalyst, as follows from the i_m_ vs. E curves of Figure 9a. In fact, the IrO_2_/white-P25 and IrO_2_/black-TiO_2_-oxy catalysts show currents that are better than those of typical state-of-the-art catalysts reported in the literature; e.g., the ATO (antimony tin oxide)-supported IrNiO_x_ catalysts of [37] exhibit a mass-specific current density of i_m_ = 90 mA/mg Ir at an overpotential of η = 280 mV, whereas our best catalysts provide the same currents at the lower overpotential of η = 240 mV. A more detailed comparison of the catalytic behavior of the materials reported in this work and some state-of-the-art catalysts in the literature is given in Table 4, which presents Ir mass-specific currents for OER at given overpotentials and was constructed by quoting or estimating values based on data reported in the referenced literature and in this work (corrected for IR-drop).

The increased mass-specific activity of the prepared catalysts of this work with respect to the commercial catalyst (despite the latter having a larger apparent esa) is due to their higher intrinsic activity as highlighted by the curves of Figure 9b, where currents are normalized by the IrO_x_ charge (esa descriptor). The higher activity of IrO_2_/TiO_2_ with respect to the unsupported IrO_2_ may be interpreted (in a way similar to the effect of Ni on IrO_2_ [41]) by the difference in electronegativity and size of Ir and Ti, which may destabilize the Ir-OH bonds and increase the presence of activated O species that are intermediates in the OER mechanism. Also, Ir-O-Ir bonds may be disrupted by the formation of Ir-O-Ti bonds as suggested in [20] based on XPS data (in a way similar to the formation/partial decomposition of Ir-O-Ni bridges proposed in [42]). As suggested in [43], XPS analysis of the O 1s is crucial in determining the presence of oxygenated species that are key to OER at oxide electrodes; such measurements, carried out by our group on similar IrO_2_/TiO_2_ samples, have shown the existence of significant quantities of OH species on the surface of the catalyst [28].

## 4. Experimental Section

### 4.1. Synthesis of IrO_2_ on Various TiO_2_ Powders

*Preparation of black-P25:* Black-P25 was produced by ammonolysis. In a typical process, ~100 mg of commercially available white-P25 (Sigma-Aldrich Chemie GmbH, Taufkirchen, Germany) powder was loaded on a crucible and placed inside a quartz tube furnace. The tube ends were sealed, and the air inside was fully replaced by ammonia gas (NH_3_ flushing). After sufficient flushing, the ammonia flow was significantly reduced and set at a constant flow rate. Then, the white-P25 was treated under ammonia at 100 °C, 300 °C, 500 °C, and 650 °C, staying at each temperature step for a total of 30 min (10 min reaching the set point temperature and 20 min for conditioning). After the last step at 650 °C, the TiO_2_ P25^®^ powder was treated there for 3 h and then freely cooled down to room temperature. 

*Preparation of TiO_2_ from hydrated TiOSO_4_ (white-TiO_2_-oxy):* Titanium (IV) oxysulfate hydrate (TiOSO_4_·xH_2_O) purchased from Sigma Aldrich, Taufkirchen, Germany, was used to prepare the *white-TiO_2_-oxy* by a co-precipitation method. Titanium dioxide was precipitated at pH ~7 from an aqueous solution of TiOSO_4_ titanium (IV) oxysulfate hydrate (0.1 M) by the addition of ammonia (VWR Chemicals, Lutterworth, UK). After aging overnight, the suspension was filtered with distilled water until it became sulfate- and ammonium ion-free and dried in air at 100 °C. The residue was crushed to a fine powder and calcined in a furnace at 700 °C for 3 h with a heating ramp rate of 5 °C min^−1^. 

*Preparation of black TiO_2_ black oxysulfate (black-TiO_2_-oxy):* The powder was produced by ammonolysis, as previously described. 

*Photodeposition of iridium nanoparticles on various TiO_2_ supports:* A photodeposition method was used for the preparation of supported OER catalysts. It is a simple and ambient temperature process, leading to the direct deposition of iridium dioxide onto the TiO_2_ semiconducting powders by UV light irradiation, as described in our previous work [28]. An appropriate iridium salt aqueous solution of 120 mL (0.002 M K_3_IrCl_6_, Sigma-Aldrich Chemie GmbH, Taufkirchen, Germany), adjusted to pH = 11 (using 1 M KOH), was prepared and deaerated under N_2_ purge for 15 min. The aqueous solution was transferred to a photochemical cylindrical reactor (250 cm^3^), and TiO_2_ powder (0.008 M) was also added. The UVA lamp (Radium Ralutec 9 W/78, λ = 350–400 nm, λ_max_ = 369 nm) was placed in a cylindrical quartz tube in the center of the photo-reactor. The materials were stirred for 20 min to prepare a homogeneous slurry. The latter was kept under UV light irradiation for 8 h to achieve (in most cases) ca 25 wt% of Ir photodeposited on the TiO_2_ nanoparticle substrate. Vacuum filtration followed with distilled water, and the resulting powder catalyst (gray, blue–black, or black in color, depending on the TiO_2_ support) was dried overnight at room temperature. The resulting solid was ground to a fine powder using an agate mortar. The four types of prepared samples were (a) IrO_2_/TiO_2_ (white-P25), (b) IrO_2_/TiO_2_ (black-P25), (c) IrO_2_/TiO_2_ (white-TiO_2_-oxy), and (d) IrO_2_/TiO_2_ (black-TiO_2_-oxy).

### 4.2. Structural, Chemical, and Morphological Measurements

The lattice parameters and the crystalline phases of the prepared TiO_2_ substrates were characterized by an X-ray Diffractometer (XRD) (Brucker AXS D8, Karlsruhe, Germany) equipped with a nickel foil monochromator (40 kV, 20 mA) at scattering angles 2θ between 10 and 80° (0.05° s^−1^ scan rate) using Cu Ka_1_ radiation. The diffraction pattern was analyzed by the Scherrer equation giving the crystallite size of each synthesized powder. The surface morphology and elemental composition were analyzed using a Field Emission Scanning Electron Microscope (FESEM; 7000 JEOL, Tokyo, Japan) with energy-dispersive X-ray spectroscopy, operating at 20 keV. Diffuse *reflectance (DR)* UV–visible spectra were measured by a Perkin Elmer Lambda 950 UV/Vis/NIR spectrophotometer working at λ = 250–2500 nm, and it was employed to measure the absorption/reflection spectrums. The optical energy bandgaps were extracted by the Tauc plot of Equation (7).

(7) $${\left(ah\nu \right)}^{n}=h\nu -E_{g}$$

where *α* is the absorption coefficient, *h* is the Planck constant, *ν* is the radiation frequency, and *n* is 0.5 or 2 for indirect or direct transition. The energy gap was also calculated at the interception of the linear fit of the appropriate region on the Tauc plot (where *α* is zero). X-ray photoelectron spectroscopy (XPS) measurements were performed using a Kratos Axis Ultra DLS spectrometer, Manchester, UK, equipped with a non-monochromatic Al X-ray source (under vacuum better than 10^−8^ Pa at a 90° take-off angle). The Ir4f line was subjected to an additional fitting procedure with XPSPEK4.1 software to determine the chemical oxidation states in the absence and presence of methanol. Transmission Electron Microscopy (TEM) images were obtained for the photocatalytically prepared powders on a JEM 2100 (JEOL, Tokyo, Japan) equipped with a LaB_6_ filament at an accelerating voltage of 200 kV. 

### 4.3. Electrochemical Measurements

Procedure: The surface electrochemistry and the electrochemical oxygen evolution activity and stability of the synthesized catalysts were studied using a PGSTAT302N potentiostat (Metrohm Autolab, Herisau, Switzerland), in a standard three-compartment glass cell equipped with a Luggin capillary and comprising a Pt foil counter electrode, a saturated calomel electrode reference electrode, and a glassy carbon (GC) working electrode substrate-current collector (3 mm diameter). The experiments were recorded in a 0.1 M HClO_4_ (70%, Sigma-Aldrich, Chemie GmbH, Taufkirchen, Germany) electrolyte at 25 °C. The system was purged with N_2_ for 15 min before the working electrode was placed in the electrolyte to remove dissolved oxygen before the electrochemical measurements were recorded. 

Electrode preparation: Prior to every measurement, the glassy carbon (GC) working electrode was polished with an alumina slurry, and then it was sonicated to remove impurities and any residual polishing slurry. Catalyst inks were coated as a thin-film layer on the GC surface of a Rotating Disk Electrode (RDE) with the resulting loading of Ir in the 0.5–1.0 mg cm^−2^ range. The ink suspension was composed of x mg catalyst (according to the *w*/*w* % Ir composition from EDS), 6% *w*/*w* Nafion^®^ perfluorinated resin solution (5 wt% Sigma-Aldrich) and ethanol as the ionic binder and solvent, respectively. It was mixed and ultrasonically homogenized for 30 min. The temperature of the sonication was kept at ca 35 °C to avoid evaporation of the solvent. 

Electrochemical characterization: The surface electrochemistry of IrO_2_/TiO_2_ was studied by cyclic voltammetry (CV) experiments in the potential range between −0.3 and 1.1 V vs. SCE at 25 mV s^−1^ until a stable CV was obtained. The last stable cycle was used to estimate the IrO_x_ charge. The OER polarization curves were recorded by Linear Sweep Voltammetry (LSV) at 1600 rpm and a slow scan rate (5 mV s^−1^). Electrochemical Impedance Spectroscopy (EIS) measurements were carried out in the frequency range of 100 kHz to 100 mHz with an amplitude of 10 mV. The ohmic resistance was estimated from the EIS measurements and use for IR compensation of the LSV curves. For the medium-term stability tests, the electrode was held at a constant potential of 1.3 V vs. SCE under a rotation speed of 1600 rpm. 

## 5. Conclusions

Summarizing, this study has demonstrated the following:

a. We showed the successful UV light photodeposition of iridium from IrCl_6_^−3^ solutions onto TiO_2_ powders in the form of IrO_x_ nanoparticles. This comprises an ambient temperature and simple chemistry alternative for the preparation of IrO_2_-TiO_2_ composites (advantages characteristic of photocatalytic synthetic methods when compared to other techniques for electrocatalyst preparation such as hydrothermal or/and sonochemical techniques; see, for example, [44,45]).
b. All of the thus prepared IrO_x_/TiO_2_ catalysts (despite a moderate apparent electroactive surface area due to electronic conductivity losses) exhibited superior mass-specific activity for OER to that of the commercial unsupported IrO_2_ catalyst, as a result of increased intrinsic activity, due to Ir-Ti interactions.
c. The best of the prepared catalysts (IrO_2_/TiO_2_ (white-P25), 26% *w*/*w* Ir, and IrO_2_/TiO_2_ (black-oxy), 28% *w*/*w* Ir), exhibited mass-specific OER activities higher than that of state-of-the-art supported IrO_x_ powder catalysts (it can be estimated as high as 100 mA mg^−1^ Ir at η = 243 mV).
d. The fact that the IrO_x_/TiO_2_ catalysts of this work outperformed IrO_x_ catalysts supported on conducting substrates (such as doped TiO_2_ and antimony-doped SnO_2_ (ATO)) indicates that the well-dispersed IrO_x_ particles anchored on TiO_2_ by the proposed photodeposition method can provide sufficient conductivity to the composite material.

## Figures and Tables

**Table 1:** EDS elemental analysis of the TiO_2_ powders before and after ammonolysis.

	Ti (%)	O (%)	C (%)	N (%)
white-P25	25.9	62.7	11.4	-
black-P25	26.8	69.6	-	3.6
white-TiO_2_-oxy	28.3	63.9	7.8	-
black-TiO_2_-oxy	28.9	68.7	-	2.4

**Table 2:** Parameters of the fitted equivalent circuit [R_s_(R_f_Q_f_)(R_ct_Q_dl_)] to EIS results, for the commercial IrO_2_, white-25, and black-TiO_2_-oxy electrodes, in 0.1 M HClO_4_ and at +1.20 V vs. SCE.

	IrO_2_	White-P25	Black-TiO_2_-Oxy
R_s_/Ω cm^2^	2.97	4.96	4.94
Q_f_/Ω^−1^s^n1^ cm^−2^	0.38	0.35	0.17
n_1_	0.78	1.09	1.10
R_f_/Ω cm^2^	0.17	0.10	0.07
Q_dl_/Ω^−1^s^n2^ cm^−2^	0.42	0.26	0.39
n_2_	0.87	0.93	0.87
R_ct_/Ω cm^2^	1.30	0.93	0.97
(C_dl_)*_Mansfeld_*/F cm^−2^	0.37	0.23	0.34
(C_dl_)*_Brug_*/F cm^−2^	0.36	0.23	0.33
R_ct_C_dl_/s *(Mansfeld)*	0.48	0.21	0.33
R_ct_C_dl_/s *(Brug)*	0.47	0.21	0.32

**Table 3:** Charge associated with 1e electrochemical oxidation of Ir in its surface oxides, as estimated from the anodic part of the CV curves of Figure 7.

	IrO_2_/White-P25	IrO_2_/Black-P25	IrO_2_/White-Oxy	IrO_2_/Black-Oxy	IrO_2_
**q_m,IrOx_/mC mg_Ir_^−1^**	85	54	94	125	199

**Table 4:** Ir mass-specific currents for OER recorded at η = 260 mV and 280 mV, for OER from acid solutions, at supported IrO_2_ nanoparticles.

Reference	Type of Catalyst	i_m_/mAmg_Ir_^−1^ (η = 260 mV)	i_m_/mAmg_Ir_^−1^ (η = 280 mV)
[38]	Ir nanodentrites/ATO	25	70
[37]	IrNiO_x_/ATO	-	90
[39]	Ir/ATO-V	30	121
[40]	IrO_2_/Nb-TiO_2_	-	82
[14]	IrO_2_-TiO_2_	10	30
[20]	IrO_2_-TiO_2_ “blue”	-	100
This work	IrO_2_/white-P25	271	877

## Data Availability

The original contributions presented in this study are included in the article; further inquiries can be directed to the corresponding author.
